# Responses of Soil Carbon Pools and Carbon Management Index to Nitrogen Substitution Treatments in a Sweet Maize Farmland in South China

**DOI:** 10.3390/plants11172194

**Published:** 2022-08-24

**Authors:** Zekai Chen, Fangdan Liu, Guangyuan Cai, Xiaoshan Peng, Xiaolong Wang

**Affiliations:** College of Agriculture, South China Agricultural University, Guangzhou 510642, China

**Keywords:** organic materials, soil organic carbon, carbon pool management index, nitrogen, sweet maize, China

## Abstract

In China, excessive nitrogen fertilizer application in sweet maize fields contributes to greenhouse gas emissions. This study used maize straw (MS), cow dung (CD), biogas residue (BR), and straw-based biochar (CB) to substitute the mineral nitrogen fertilizer at 20% and 50% ratios in the Pearl River Delta in China. In comparison with a conventional amount of mineral nitrogen fertilizer (CK), the soil organic carbon (SOC) storages of the different treatments increased by 6.5–183.0%. The CB treatment significantly improved the inert organic carbon pool in the soil, while other types of organic materials promoted the formation of activated carbon pools. The treatments increased the soil carbon pool management index by 21.1–111.0% compared to the CK. Moreover, the CB treatments increased the soil carbon sequestration index by 78.3% and 155.8% compared to the CK. In general, substituting the mineral N fertilizer with BR, CB, and CD could improve the SOC accumulation in sweet maize farmland in South China. The CB at the high substitution level was the best measure for stabilizing carbon sequestration in the sweet maize cropping system. This experiment provides valuable information for ensuring the clean production of sweet maize in a typical subtropical area in East Asia.

## 1. Introduction

Agriculture is an important source of greenhouse gas (GHG) emissions. The total amount of GHGs produced by agricultural systems was 5.41 billion tons [1], accounting for approximately 14% of the total global GHGs [2]. A large number of studies have demonstrated that the excessive application of nitrogen fertilizer was the primary cause of GHG emissions from agricultural systems [3,4]. China is the largest producer and consumer of nitrogen fertilizer in the world. China’s annual use of nitrogen fertilizer for crop production accounts for 24.9% of the world’s total [5]. The GHG emissions from the application of nitrogen fertilizer account for 53–57% of the total GHG emissions from crop production in China, making up the largest proportion [6]. Therefore, the reduction in nitrogen fertilizer application has been the key practice to reduce agricultural GHG emissions in China.

At the same time, the agricultural system functions as an important carbon sink in the terrestrial ecosystem [7]. The global technical mitigation potential for agriculture in 2030 was estimated to reach up to 0.5–10.6 billion tons CO_2_-eq each year [8]. The soil carbon pool in farmland is the most disturbed and regulated carbon pool on earth. The international community hopes to slow global climate change by increasing soil carbon sequestration. In China, the soil organic carbon (SOC) content in farmland is lower than the global average level [9]. Thus, there is a huge potential for carbon sequestration in farmland in China. Previous studies illustrated that the application of exogenous organic materials was the most long-term and effective means of soil carbon sequestration [10,11,12]. Moreover, the application of organic materials could increase soil organic matter content and improve soil structure and soil fertility [7,13]. China is a major producer of agricultural organic wastes. About 970 million tons of crop residues [14] and 3.16 billion tons of animal manure [15] are generated in China every year. The irrational use of the organic wastes was another important source of GHG emissions from agricultural fields [16]. Therefore, returning the organic materials to replace the mineral nitrogen fertilizer in farmland would not only reduce the application of mineral nitrogen input but would also increase the content of SOC in farmland. The nitrogen fertilizer substitution (NSS) practice could be an effective means for the Chinese agricultural system to decrease GHG emissions and increase the soil organic carbon content (SOC_c_) of farmland.

During the past decades, many scientists have studied the effects of the NSS practice on soil carbon sequestration in various crops in the world. For example, Di et al. [17] found that the long-term application of organic materials could promote the retention of SOC in paddy soil. Siedt et al. [18] indicated that biochar was better than straw and compost for carbon sequestration in the crop production system. Singh Brar et al. [19] found that the combined use of organic and inorganic fertilizers increased soil carbon sequestration. Cai et al. [20] suggested that the combination of mineral fertilizer and organic fertilizer could significantly increase the soil organic carbon in dryland farmland. Xu et al. [21] found that long-term straw returning could promote surface organic carbon accumulation in maize cropping systems, while an insufficient nitrogen supply led to carbon depletion in the bottom soil. In this context, more attention was paid to the composition and stability of carbon pools in recent years. Meng and Liu [22] studied the effects of organic materials on different SOC components, indicating that the bio-organic fertilizer significantly increased the dissolved organic carbon (DOC) and easily oxidized organic carbon (ROC) and microbial carbon contents (MBC). Sodhi et al. [23] calculated the long-term soil active carbon content and carbon pool management index of a rice–wheat rotation, demonstrating a higher soil active carbon content under a rice straw composting treatment. Das et al. [24] suggested that the long-term application of organic fertilizer could significantly improve the soil organic carbon content and soil aggregate stability in a rice–wheat rotation system. Moharana et al. [25] found that organic materials (rice straw, mustard stover, and leaves) combined with fertilizer improved the soil carbon pool management index and the soil organic carbon content in a wheat–green gram cropping system. Yang et al.’s study [26] showed that biochar application not only significantly improved soil quality by increasing organic carbon components but also improved soil carbon pool stability by increasing inert organic carbon (NLC). Clearly, the composition and stability of the soil carbon pools in different crop–soil systems were not completely the same under different agricultural practices.

Sweet maize (Zea mays L. saccharata) is a characteristic crop that originated in the United States and was introduced in countries around the world with increasing popularity as a favored choice [27]. Sweet maize has a huge market potential. At present, China is the second largest producer of sweet maize [28]. The main planting area of sweet maize in China is Guangdong province, with a total planting area of 400,000 hectares [1]. In this region, the sweet maize is mostly planted at 2 to 3 crops per year under the suitable hydrothermal conditions [29]. Due to the high nitrogen demand and good economic benefits of sweet maize cultivation, farmers often apply excessive nitrogen fertilizer in the planting process of sweet maize [30,31]. As a result, the applied quantity of mineral nitrogen fertilizer each year in sweet maize farmland is generally higher than other grain crop cultivation systems in South China. In this context, the cultivation of sweet maize has been an important source of agricultural GHG emissions in South China. The application of NSS practice in the sweet maize cropping system in South China showed a huge potential to decrease the GHG emissions and increase the soil carbon sequestration [19,20,31]. However, information on the effects of NSS practice on soil carbon sequestration in sweet maize fields is still very limited.

Therefore, this study compared different NSS practices in a sweet maize in the Pearl River Delta in China based on field research. The responses of soil carbon pools and the carbon management index to different NSS treatments in a sweet maize farmland were analyzed. The objective of the study was to screen out the suitable NSS practice that contributed to increasing the SOC_c_ and to improving the stability of the soil carbon pool in a sweet maize farmland in South China. The study could provide a research foundation for the establishment of a low-carbon farming system in the subtropical area in East Asia.

## 2. Results

### 2.1. Soil Organic Carbon Accumulation

As shown in Table 1, the soil bulk density of TBR, TCB, and TMS decreased by 3.7%, 8.1%, and 0.7%, respectively, compared to the CK under the low-substitution-ratio (SR) treatments, but the differences were not significant. For the SOC_c_, only the TCB increased by 103.1% compared with the CK, with a significant difference. As a result, the soil organic carbon storages (SOC_s_) of the treatments were 6.5–86.8% higher than the CK. Therein, the TCB showed a significant difference with the CK. The results showed that the soil organic carbon accumulation could be improved by the NSS practice under the low-SR condition, but only the TCB showed a significant difference with the CK.

In the high-SR experiment, the soil bulk density of FBR, FCB, FCD, and FMS decreased by 4.4–21.3%, respectively, compared to the CK. Therein, the FCB and FCD showed significant differences. Meanwhile, except for the maize straw (MS) treatment, the SOC_c_ values of FBR, FCB, and FCD were 82.1%, 259.0%, and 79.2% higher than the CK, with significant differences. Similarly, compared to the CK, the SOC_s_ of FBR, FCB, and FCD increased by 70.3%, 183.0%, and 60.2%, respectively, with significant differences. The results showed that when nitrogen fertilizer was substituted by organic materials with high quantities, the SOC_s_, except FMS, could be significantly increased.

### 2.2. Soil Organic Carbon Components and Carbon Sequestration Index in the Low-SR Experiment

#### 2.2.1. Soil Organic Carbon Components

**Figure 1:**
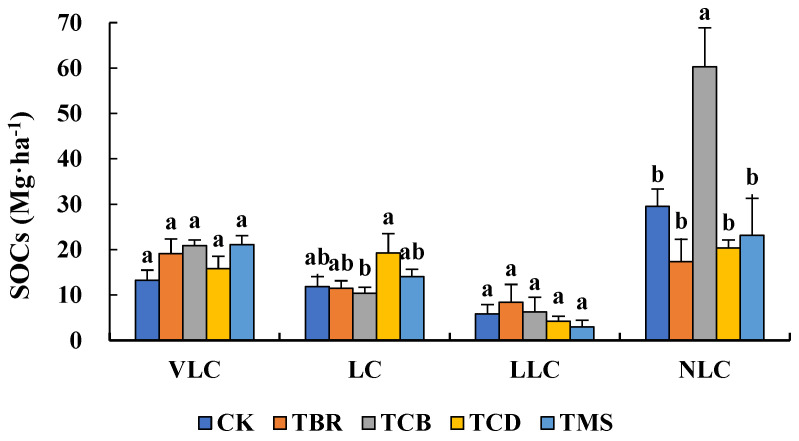
Soil organic carbon storage (SOC_s_) of each component (Mg·ha^−1^) under low-SR treatment. Small letters denote significant differences between treatments at P < 0.05.

According to Figure 1, the highly active organic carbon (VLC) in TBR, TCB, TCD, and TMS increased by 44.3%, 57.3%, 19.0%, and 59.1%, but the differences were not significant. For the active organic carbon (LC) and intermediate active organic carbon (LLC), TCD and TMS increased the content of LC by 63.0% and 18.5% and decreased the content of LLC by 27.6% and 49.0%. Meanwhile, the TBR and TCB decreased the content of LC by 0.2% and 12.2% and increased the content of LLC by 42.8% and 7.2%. However, there was no significant difference in LC content between TCB and TCD. For the inert organic carbon (NLC), TCB increased by 104.1%, with a significant difference compared to the CK. The NLC content in TBR, TCD, and TMS were 41.4%, 31.2%, and 21.6% lower than the CK, without significant differences.

#### 2.2.2. Soil Carbon Sequestration Index

As shown in Table 2, compared with the CK, the active organic carbon pool (AC) in TBR, TCB, TCD, and TMS increased by 22.1%, 24.6%, 40.0%, and 40.0%, respectively, in which the TCD and TMS showed significant differences with the CK. Conversely, only the inert organic carbon pool (PC) in TCB was higher than the CK with a significant difference. As a result, the TCB significantly increased the soil carbon sequestration index (RI) by 78.3% compared to the CK, with a significant difference, while the RIs of TBR, TCD, and TMS decreased by 24.2–39.2%. The results showed that the NSS practice in the low-SR condition could improve the AC in the soil, except for the application of straw-based biochar.

### 2.3. Soil Organic Carbon Components and Carbon Sequestration Index in the High-SR Experiment

#### 2.3.1. Soil Organic Carbon Components

**Figure 2:**
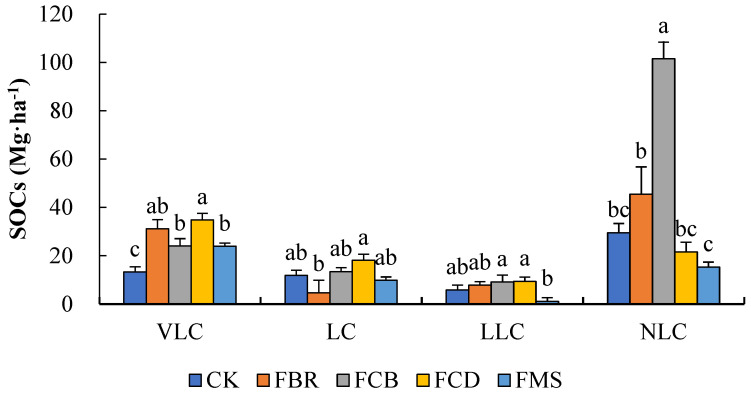
Soil organic carbon storage (SOC_s_) of each component (Mg·ha^−1^) under high-SR treatment. Small letters denote significant differences between treatments at P < 0.05.

As shown in Figure 2, the VLC values of FBR, FCB, FCD, and FMS were significantly increased by 134.7%, 81.2%, 162.4%, and 80.2% compared to the CK. For the LC, the FCB and FCD were 13.6% and 53.0% higher, while the FBR and FMS were 61.1% and 16.9% lower than the CK, but the differences were not significant. The LLC of FMS decreased by 81.1%, while the values in FBR, FCB, and FCD increased by 33.1%, 56.1%, and 60.6% compared to the CK. For the NLC, only the FCB was 243.8% higher than the CK, with a significant difference.

#### 2.3.2. Soil Carbon Sequestration Index

As shown in Table 3, compared with the CK, the AC in FBR, FCB, FCD, and FMS increased AC by 43.1%, 49.4%, 110.9%, and 34.4%, among which the FCB and FCD showed significant differences. The PC of FMS decreased by 43.5%, but the difference was not significant. Conversely, the PC in FBR, FCB, and FCD increased by 95.4%, 305.9%, and 13.6%, indicating a significant difference in FCB. As a result, the RI values in FBR and FCB were 33.3% and 155.8% higher than the CK, while the index values in FCD and FMS were 48.3% and 61.7% lower. Therein, only the FCB showed a significant difference compared to the CK. The results showed that a large replacement of organic materials could improve the AC, while FBR and FCB could improve soil stability and carbon sequestration.

### 2.4. Effects of Nitrogen Replacement on Soil Carbon Pool Management Index

Table 4 presents the carbon pool management index (CMI) of different treatments. In the low-SR experiment, the CMIs in TBR, TCB, TCD, and TMS increased by 21.1%, 26.1%, 22.2%, and 32.7%, respectively, compared to the CK, but the differences among them were not significant. However, the CMIs in FBR, FCB, FCD, and FMS were 49.5%, 52.1%, 111.0%, and 27.7% higher, respectively, than the CK in the high-SR experiment. Except for the FMS, the other treatments showed significant differences compared to the CK.

### 2.5. Effect of Substitution Ratios

**Figure 3:**
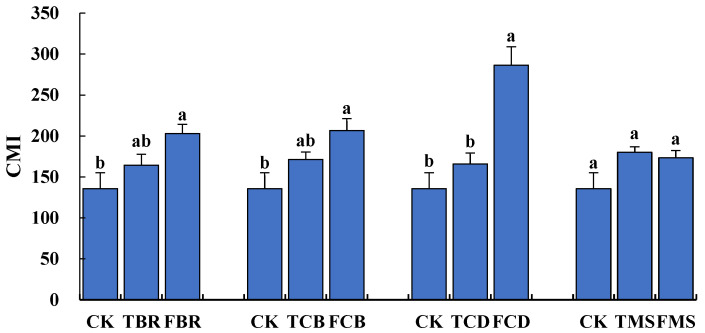
Effects of different degrees of nitrogen replacement on carbon management index (CMI). Small letters denote significant differences between treatments at P < 0.05.

Figure 3 shows the changes in different NSS practices in the low- and high-SR conditions. Clearly, compared with the CK, the CMIs of biogas residue (BR), straw-derived biochar (CB), and cow dung (CD) based NSS practice treatments showed increasing trends with the increased SR. Moreover, the three NSS practices showed significant differences when the SR was 50%. It should be noted that the MS-based NSS practice showed a lower CMI at the high-dose SR than the low-dose one, but the difference between them was not significant.

## 3. Discussion

### 3.1. Effects on SOC Accumulation Derived from Different NSS Practices

This study demonstrated that returning different organic materials to the field contributed to improving the SOC in sweet maize farmland in South China. Similarly, Zhang et al. [32] found that organic amendments had significant effects on soil carbon sequestration in paddy fields in subtropical regions as alternative and supplementary nutrients. Kowalska et al. [12] believes that biofertilizers help improve soil quality and carbon sequestration. Tang et al. [33] found that biogas slurry could replace chemical fertilizer to improve the organic carbon storage of a straw returning system. Clearly, the results in the present research were consistent with the published papers. On one hand, organic materials contain a large amount of lignin, which protects the soil carbon source and prolongs the existence of carbon in the soil [34]. On the other hand, long-term application of nitrogen fertilizer changed the activities of cellulose- and lignin-degrading enzymes, delayed the decomposition [35], and formed more stubborn lignin-like compounds [36]. At the same time, most of the carbon in organic materials exists in a form that is difficult to degrade and has been decomposed to a certain extent before application in the agricultural field, resulting in more carbon sequestration [23]. It should be noted that the straw returning in the high-SR experiment in the present study did not increase SOC compared to the CK. Zhang et al. [37] and Ding et al. [38] also found that there was little change in the surface soil organic carbon in black soil after long-term maize straw returning. The reason may be that, compared with other organic materials, the crushed straw lacks a decomposition process. The organic carbon in straw cannot be transformed into a form that is difficult to degrade, thus playing a protective role. In addition, the application of a small amount of nitrogen fertilizer may have a weak retarding effect on lignin decomposition, resulting in a straw mineralization rate that may be greater than the formation rate of soil organic carbon. Moreover, the planting system may be affected by the experimental years and has not reached the organic carbon balance [19], which should be further studied in the future.

### 3.2. Effects on SOC Stability Derived from Different NSS Practices

Returning organic materials to the field has a certain effect of carbon enhancement, and it usually increases more in the activated organic carbon pool. This characteristic is beneficial to the nutrient supply and yield improvement of crops. Related studies have also shown the effect on production increase [39,40,41,42]. However, this study indicated that the contribution of organic materials to PC was small, although the total SOC_s_ increased. In other words, the NSS practices would not be conducive to stable carbon sequestration in soil, except for the biochar-based NSS practice. Biochar is beneficial to soil stability and carbon sequestration. The effects of biochar have been reported in many relevant studies in past years [18,43,44]. Due to the low content of easily oxidized organic carbon and dissolved organic carbon in soil modified by biochar, returning biochar into farmland always causes low bioavailability and the inhibition of the mineralization of organic matter [43]. Compared to the biochar-based NSS practices, returning other organic materials to the field always resulted in increased microbial carbon contents (MBC), easily oxidized organic carbon (ROC), and dissolved organic carbon (DOC) [45,46]. Presently, biochar is usually viewed as a carbon sink with considerable potential, playing an important role in soil carbon sequestration and offsetting increased atmospheric CO_2_ concentrations [47]. This study also demonstrated that the biochar-based NSS practice was indeed the best measure for stable carbon sequestration in sweet maize farmland in South China if we did not consider crop productivity.

### 3.3. Effect of Substitution Ratio

This study found that the returning effects of organic materials with high-SR were generally better than those with low-SR with regards to soil carbon sequestration. Except for the MS treatment, the SOC_s_ increased by 44.4–60.0% when the replacement of other organic materials with high-volume was higher than that with low-volume. In addition, compared with a low-volume replacement of organic materials, a high-volume replacement of organic materials increased CMI by 20.6–72.6%. Luan et al. [48] found that 75% manure replacement reduced BD by 7.4% and increased SOC_c_ by 31.6% compared with 25% manure replacement. Shi et al. [49] found that 40% biochar substitution increased SOC_c_ by 8.8% and DOC by 30.4% compared with 20% biochar substitution in yellow paddy soil. Zhu et al. [50] found that the SOC_s_ increased by 12.0%, 43.2%, 69.3%, and 71.2% with the increase in nitrogen replacement rates (30%, 50%, 70%, and 100%) in the North China Plain. The results in the published studies were consistent with those in the present case. A higher SR in NSS practices represented a lower application of mineral nitrogen fertilizer, which tended to lead to mineralization and the loss of soil organic matter [51,52]. Ammonium nitrate and urea-containing fertilizer significantly accelerated the release of total C from decayed organic matter, while organic fertilizer slowed the release of total C from decayed organic matter [34]. That was one reason illustrating that the high-SR caused high SOC accumulation and CMIs. Another reason was that the higher application amount of exogenous carbon in the high-SR experiment was artificially inputted into the farmland. However, the SOC and CMI of the MS treatment in the high-SR experiment did not show a significant difference compared to the low-SR experiment in this study. The reason may be that the crushed straw was not decomposed and the lignin content was high, which further inhibits the straw utilization efficiency. A longer-term experiment will be required to illustrate the results in the future.

## 4. Materials and Methods

### 4.1. Study Site

The field experiment was conducted at an experimental farm of South China Agricultural University in Guangzhou city in China (24°14′ N, 113°38′ E). The region has a subtropical monsoon climate with an average annual temperature of 23.6 °C, a frost-free period of 335−360 days, and an average annual precipitation of 1810 mm. This study was conducted from June 2020 to August 2021, including four sweet maize growing seasons.

### 4.2. Experiment Design

This study included two experiments on different substitution ratios (SR) of mineral nitrogen fertilizer by using different types of organic materials, including a high-SR (50%) and a low-SR (20%). In each SR experiment, we selected four types of organic materials, including maize straw (MS), straw-derived biochar (CB), biogas residue (BR), and cow dung (CD), to replace chemical N fertilizer in a sweet maize cropping system, which were the common agriculture-derived wastes in the study region. The MS was derived from the maize system itself. The CB was processed from maize straw under a high temperature. The CD was a by-product of animal husbandry. The BR was a by-product of a biogas project after fermenting animal manure. The four types of organic materials were applied in the sweet maize system to replace the N fertilizer based on a principle of equal N input. Each experiment was arranged in a completely randomized block design with five treatments and three replicates. The area of each cropping plot was about 28 m^2^ (4.0 m × 7.0 m). The applied amounts of organic materials were determined according to their N contents (Table 5). The total nitrogen contents of the organic materials were measured before the application of the organic materials (Table 6). The initial soil properties of the high-SR experiment were 5.8 g·kg^−1^ SOC, 1.18 g·kg^−1^ total N, 1.59 g·kg^−1^ total P, 4.38 g·kg^−1^ total K, and a pH of 5.79. The initial soil properties of the low-SR experiment were 17.8 g·kg^−1^ SOC, 1.01 g·kg^−1^ total N, 1.15 g·kg^−1^ total P, 1.47 g·kg^−1^ total K, and a pH of 5.56.

### 4.3. Field Management

The field management of this experiment was consistent with the local sweet maize farms, expect for the fertilization practice. The cultivar of sweet maize used in the study was “Huameitian 48”, which was the main variety applied in Guangdong province in China. The sweet maize seedlings were raised in the seedling tray and transplanted at the three-leaf stage. Ridge cultivation was used in the field. Before maize transplanting, a small rotary tiller was used to plow the farmland, and the furrow was leveled manually. The ridge spacing, width, and height were 125 cm, 100 cm, and 25 cm, respectively. Two rows of maize were transplanted on the ridges with a hole spacing of 30 cm. The sweet maize was supplied with N fertilizer as urea. All treatments were supplied with 150 kg·ha^−1^ of P fertilizer (as calcium superphosphate) and 150 kg·ha^−1^ of K fertilizer (as potassium chloride). The chemical fertilizers were divided into four proportions, 30%, 35%, 30%, and 5%, and were applied as seed dressing, jointing dressing, attacking bud dressing, and strong grain dressing, respectively. The organic materials were evenly spread as a base fertilizer by manual work before ridging and were turned into the soil by a ploughing machine. The depth should have been 10–15 cm in the soil layer, and the mixture should have been evenly mixed. Among them, maize straw needed to be cut up with a grass cutter and then evenly pressed into the plot. During the crop growing period, pesticide management was carried out in accordance with the situation of the field weeds and pests. Irrigation by mechanical power was carried out in the main growth period of sweet maize. The maize straw was removed from the field after harvest.

### 4.4. Data Measurement

#### 4.4.1. Soil Sampling

Soil collection was conducted in August, after the sweet maize harvest in the summer of 2021, to reflect the characteristics of soil carbon storage after four continuous seasons of experiments. Random multipoint sampling was carried out in each plot, and samples were taken from each plot. In each sampling, 0−10 cm and 10−20 cm soil were taken from the same collection point. Then, the layers of soil were mixed into a sample of one point. Finally, the samples from the two points in each plot were mixed into one sample. The visible roots, stems, leaves, insect bodies, stones, and nodules were picked out. After drying, the samples were ground through a 0.15 mm sieve.

#### 4.4.2. Determination of Soil Bulk Density and Organic Carbon Content

The soil bulk density was measured by the ring knife method [53]. The total SOC was determined by the potassium dichromate oxidation-external heating method (Walkley and Black 1934). The composition of the SOC was differentiated based on the degree of oxidation of different components according to the modified Walkley–Black oxidation method [54]. The organic carbon content determined by 6 mol·L^−1^ H_2_SO_4_ was determined as VLC, the difference between 6 and 9 mol·L^−1^ H_2_SO_4_ was determined as LC, the difference between 9 and 12 mol·L^−1^ organic carbon content determined by H_2_SO_4_ was intermediate LLC, and the difference in the total organic carbon content determined by 12 mol·L^−1^ H_2_SO_4_ was NLC.

### 4.5. Indicators

#### 4.5.1. Soil Organic Carbon Storage

The organic carbon content (SOC_c_) obtained in the test measurement was converted into SOC storage (SOC_s_) by the Equation (1):

(1) $${\mathrm{SOC}}_{s}={\mathrm{SOC}}_{c}\times \mathrm{BD}\times D\times 10$$

where SOC_s_ and SOC_c_ represent the SOC storage (Mg·ha^−1^) and the SOC content (g·kg^−1^), respectively; BD is the soil bulk density (Mg·m^−3^); D represents the soil depth, which was 0.20 m in this study; and the 10 represents the conversion coefficient.

#### 4.5.2. Soil Active Carbon Pool, Soil Inert Carbon Pool, and Soil Carbon Sequestration Index

The SOC pool was divided into AC and PC, which were calculated by Equations (2) and (3), respectively. Then, the RI was calculated to explore the impact of agricultural management measures on the soil carbon sequestration capacity [55] according to Equation (4):

(2) AC=∑(VLC+LC)

(3) PC=∑(LLC+NLC)

(4) RI=PC∕AC

where AC and PC represent active organic and inert organic carbon pools (Mg·ha^−1^), respectively and VLC, LC, LLC, and NLC represent high, medium, low, and inert organic carbon pools (Mg·ha^−1^), respectively.

#### 4.5.3. Soil Carbon Pool Management Index

The CMI was calculated in this study to quantitatively evaluate the stability of the soil carbon pool based on Equations (5)–(7) [56,57]:

(5) CMI=CPI×LI×100

(6) $$\mathrm{CPI}={\mathrm{TOC}}_{i}∕{\mathrm{TOC}}_{\mathrm{CK}}$$

(7) LI=(VLC∕TOC)×3+(LC∕TOC)×2+(LLC∕TOC)×1

where the CPI and LI are carbon pool index and carbon pool activity index, respectively; *i* represents the alternative treatments of different organic materials in the experimental groups; and CK represents the control treatment.

### 4.6. Statistical Analysis

The experimental results were sorted and statistically analyzed by Excel 2019 and SPSS Statistics 26, respectively. The data were tested by Duncan’s multiple comparison method (P < 0.05).

## 5. Conclusions

This study demonstrated that the NSS practice could increase the SOC by 6.5–183.0% and CMIs by 21.1–111.0% in sweet maize farmland in South China. The CB treatments in the low- and high-SR experiments both significantly increased the SOC in top soil, while the BR and CD treatments showed significant differences compared to the CK only in the high-SR experiment. From the composition of the soil carbon pool, only the CB treatment significantly improved the inert organic carbon pool in the soil, while the other types of organic materials promoted the formation of an activated carbon pool. Moreover, this study indicated the high-SR of the NSS practices showed higher SOC_s_ and CMIs than the low-SR. In addition, the CB treatments in the low- and high-SR experiments significantly increased the RI by 78.3% and 155.8%, respectively, compared to the CK. The RI of the BR treatment in the high-SR experiment was 33.3% higher than the CK, with a significant difference, while the other treatments decreased the RI by 24.2–61.7%. Generally, this study found that substituting the mineral N fertilizer by returning the biogas residue, biochar, and cow dung to the field contributed to improving the SOC accumulation in sweet maize farmland in South China, especially at high-SR. In the different types of organic materials, the biochar-based NSS practice was the best measure for stable carbon sequestration in sweet maize farmland in South China. This experiment provided valuable information for constructing a low-carbon farming system for sweet maize production in a typical subtropical area in East Asia.

## Figures and Tables

**Table 1:** Soil bulk density (BD), soil organic carbon content (SOC_c_), and soil organic carbon storage (SOC_s_) under different treatments in the study.

Experiment	Treatments	Bulk Density (Mg·m^−3^)	SOC_c_ (g·kg^−1^)	SOC_s_ (Mg·ha^−1^)
Low-SR experiment	Conventional N (CK)	1.36 ± 0.002 a	19.23 ± 1.77 b	52.36 ± 4.83 b
	50% N + Maize straw (TMS)	1.31 ± 0.043 a	21.31 ± 0.65 b	55.75 ± 1.69 b
	50% N + Biochar (TCB)	1.25 ± 0.058 a	39.06 ± 4.26 a	97.82 ± 10.67 a
	50% N + Biogas residue (TBR)	1.36 ± 0.024 a	21.28 ± 0.63 b	58.07 ± 1.73 b
	50% N + Cow dung (TCD)	1.35 ± 0.029 a	22.69 ± 3.22 b	61.26 ± 8.70 b
High-SR experiment	Conventional N (CK)	1.36 ± 0.002 a	19.23 ± 1.77 c	52.36 ± 4.83 c
	20% N + Maize straw (FMS)	1.27 ± 0.056 ab	35.01 ± 4.22 b	89.19 ± 10.76 b
	20% N + Biochar (FCB)	1.07 ± 0.065 c	69.03 ± 3.56 a	148.19 ± 7.63 a
	20% N + Biogas residue (FBR)	1.22 ± 0.080 b	34.46 ± 2.03 b	83.88 ± 4.94 b
	20% N + Cow dung (FCD)	1.30 ± 0.024 ab	18.95 ± 0.35 c	49.12 ± 0.91 c

Data are shown as means ± standard errors. Different lowercase letters after numbers in the same column indicate significant differences at P < 0.05 using Duncan’s test.

**Table 2:** Soil activity, inert organic carbon pools (AC and PC), and carbon sequestration index (RI) under low-substitution-ratio experiment.

Treatment	AC (Mg·ha^−1^)	PC (Mg·ha^−1^)	RI
CK	25.08 ± 3.06 b	27.28 ± 3.71 b	1.20 ± 0.58 b
TBR	30.63 ± 3.12 ab	25.12 ± 2.66 b	0.91 ± 0.44 b
TCB	31.24 ± 1.70 ab	66.58 ± 10.17 a	2.14 ± 0.86 a
TCD	35.05 ± 3.13 a	23.02 ± 2.47 b	0.74 ± 0.46 b
TMS	35.10 ± 0.77 a	26.16 ± 8.19 b	0.73 ± 0.53 b

Different lowercase letters after numbers in the same column indicate significant differences at P < 0.05 using Duncan’s test.

**Table 3:** Soil activity, inert organic carbon pools (AC and PC), and carbon sequestration index (RI) under high-substitution-ratio experiment.

Treatment	AC (Mg·ha^−1^)	PC (Mg·ha^−1^)	RI
CK	25.08 ± 3.06 c	27.28 ± 3.71 bc	1.20 ± 0.58 bc
FBR	35.89 ± 2.16 bc	53.31 ± 12.20 b	1.60 ± 1.14 b
FCB	37.46 ± 3.07 b	110.72 ± 7.82 a	3.07 ± 0.87 a
FCD	52.89 ± 4.78 a	30.99 ± 3.25 bc	0.62 ± 0.27 bc
FMS	33.71 ± 0.60 bc	15.41 ± 1.38 c	0.46 ± 0.12 c

Different lowercase letters after numbers in the same column indicate significant differences at P < 0.05 using Duncan’s test.

**Table 4:** Soil carbon pool index (CPI), carbon pool activity index (LI), and carbon pool management index (CMI) under different SR experiments.

Experiment	Treatment	CPI	LI	CMI
Low-SR experiment	CK	1.00 ± 0.00 c	1.36 ± 0.19 ab	135.76 ± 19.25 ab
	TBR	1.06 ± 0.03 c	1.55 ± 0.13 a	164.43 ± 13.20 a
	TCB	1.87 ± 0.20 a	0.96 ± 0.08 bc	171.14 ± 9.27 a
	TCD	1.11 ± 0.03 c	1.49 ± 0.08 ab	165.92 ± 13.13 a
	TMS	1.17 ± 0.17 bc	1.65 ± 0.16 a	180.09 ± 6.76 a
High-SR experiment	CK	1.00 ± 0.00 d	1.36 ± 0.19 bc	135.76 ± 19.25 c
	FBR	1.70 ± 0.21 b	1.29 ± 0.17 bc	202.90 ± 11.24 b
	FCB	2.83 ± 0.15 a	0.73 ± 0.05 a	206.46 ± 14.81 b
	FCD	1.60 ± 0.09 bc	1.79 ± 0.10 c	286.45 ± 22.52 a
	FMS	0.94 ± 0.02 d	1.86 ± 0.12 c	173.34 ± 8.97 bc

Different lowercase letters after numbers in the same column indicate significant differences at P < 0.05 using Duncan’s test.

**Table 5:** Type and amount of applied fertilizer in the study (kg·ha^−1^).

Experiment	Treatment	Mineral Fertilizer Inputs (kg·ha^−1^)			Organic Materials (kg·ha^−1^) *	N Input (kg·ha^−1^)
		N	P_2_O_5_	K_2_O		
High-SR experiment	CK	300	150	300	0	300
	TBR	150	150	300	27,227	300
	TCB	150	150	300	30,086	300
	TCD	150	150	300	46,617	300
	TMS	150	150	300	45,966	300
Low-SR experiment	CK	300	150	300	0	300
	FBR	240	150	300	10,891	300
	FCB	240	150	300	12,035	300
	FCD	240	150	300	18,647	300
	FMS	240	150	300	18,386	300

* The applied amounts of the organic materials presented here were their average values during the experimental period.

**Table 6:** Total nitrogen content (%) of applied organic materials in each crop growing season in the study.

Organic Material	2020 Summer	2020 Autumn	2021 Spring	2021 Summer
Cow dung	0.81%	0.60%	1.50%	1.88%
Biogas residue	1.07%	1.51%	1.22%	2.57%
Maize straw	1.16%	1.02%	1.38%	1.62%
Straw-derived biochar	0.51%	0.56%	0.86%	0.83%

## Data Availability

Not applicable.
